# Measurement of language laterality using functional transcranial Doppler ultrasound: a comparison of different tasks

**DOI:** 10.12688/wellcomeopenres.14720.3

**Published:** 2020-02-11

**Authors:** Zoe V.J. Woodhead, Holly A. Rutherford, Dorothy V.M. Bishop

**Affiliations:** 1 Department of Experimental Psychology, University of Oxford, Oxford, UK

**Keywords:** Language, lateralization, hemispheric dominance, functional transcranial Doppler sonography (fTCD), speech production, verbal fluency

## Abstract

**Background:** Relative blood flow in the two middle cerebral arteries can be measured using functional transcranial Doppler sonography (fTCD) to give an index of lateralisation as participants perform a specific task. Language laterality has mostly been studied with fTCD using a word generation task, but it is not clear whether this is optimal.

**Methods: **Using fTCD, we evaluated a sentence generation task that has shown good reliability and strong left lateralisation in fMRI. We interleaved trials of word generation, sentence generation and list generation and assessed agreement of these tasks in 31 participants (29 right-handers).

**Results**: Although word generation and sentence generation both gave robust left-lateralisation, lateralisation was significantly stronger for sentence generation. Bland-Altman analysis showed that these two methods were not equivalent. The comparison list generation task was not systematically lateralised, but nevertheless laterality indices (LIs) from this task were significantly correlated with the other two tasks. Subtracting list generation LI from sentence generation LI did not affect the strength of the laterality index.

**Conclusions**: This was a pre-registered methodological study designed to explore novel approaches to optimising measurement of language lateralisation using fTCD. It confirmed that sentence generation gives robust left lateralisation in most people, but is not equivalent to the classic word generation task. Although list generation does not show left-lateralisation at the group level, the LI on this task was correlated with left-lateralised tasks. This suggests that word and sentence generation involve adding a constant directional bias to an underlying continuum of laterality that is reliable in individuals but not biased in either direction. In future research we suggest that consistency of laterality across tasks might have more functional significance than strength or direction of laterality on any one task.

## Background

It is well-established that language is predominantly processed in the left cerebral hemisphere in the majority of people. The functional significance of this bias is not understood, and it has been difficult to study the impact of individual variation because, on the one hand, those with atypical lateralisation are relatively rare (estimated as around 5 per cent of right-handers, Mazoyer *et al.*, 2014), and on the other hand, assessment of cerebral lateralisation in non-clinical populations is time-consuming and challenging with large samples.

### Measuring lateralisation with Functional Transcranial Doppler ultrasonography (fTCD)

Functional transcranial Doppler ultrasonography (fTCD) is a minimally invasive technique which is portable and relatively inexpensive, making it possible to investigate hemispheric dominance in relation to typical and impaired language function across a wider population. This method measures bilateral blood flow in the middle cerebral arteries (MCA) – which may be regarded as a proxy of cerebral activity in regions supplied by these vessels due to neurometabolic coupling ( Lohmann *et al.*, 2006). Although the territory of the MCA is variable between individuals, it consistently includes the key language regions of Broca’s and Wernicke’s areas ( Deppe *et al.*, 2004). In addition to being mobile and inexpensive, fTCD is largely unaffected by head movements and muscle artefacts during speech ( Gutierrez-Sigut *et al.*, 2015), meaning large numbers of participants can be investigated – including children and those who are unsuitable for other recording methods. The principal drawback of fTCD is that, while it is reliable at detecting increased blood flow in one hemisphere during an activation task with good temporal resolution, it cannot be used to localise function within a hemisphere.

### Language tasks use to assess language lateralisation

The word generation task ( Knecht *et al.*, 1998a; Knecht *et al.*, 1998b) has been used as the gold standard method for assessing cerebral lateralisation using fTCD – with good concordance with fMRI ( Deppe *et al.*, 2000; Somers *et al.*, 2011) and Wada technique ( Knecht *et al.*, 1998a; Wada & Rasmussen, 1960), and adequate test-retest reliability ( Knecht *et al.*, 1998a). Although other tasks have been shown to be sensitive and reliable at assessing cerebral lateralisation, we still know relatively little about the key characteristics of tasks that give reliable left lateralisation. Indeed, it has been proposed that language laterality is multifactorial, and may dissociate within an individual from one task to another ( Gaillard *et al.*, 2004; Stroobant *et al.*, 2009; Tailby *et al.*, 2017).

### Use of comparison tasks in fTCD and fMRI

One difference between fTCD and fMRI is that the latter typically includes a comparison task against which a language activation task is assessed. (We use here the term ‘comparison task’ rather than the more customary ‘baseline task’, to avoid confusion, as the term ‘baseline’ is used in fTCD to refer to the rest period prior to the language activation period). Subtracting activation from a comparison task is used in fMRI to remove activation associated with incidental aspects of task processing. For instance, if the task involves describing a picture, then a comparison task may involve viewing, but not describing, a picture, with the goal of removing activation associated with picture-viewing. Choice of comparison task in fMRI can have a substantial impact on the laterality index ( Bradshaw *et al.*, 2017); for example, Binder and colleagues ( Binder *et al.*, 2008) observed that laterality indices were stronger when language tasks were contrasted with active comparison tasks. In fTCD, left and right channels are mathematically equated by subtracting the levels during a baseline rest period from the whole trial, but the impact of subtracting the timecourse of a comparison task has not yet been ascertained.

**Figure 1.**
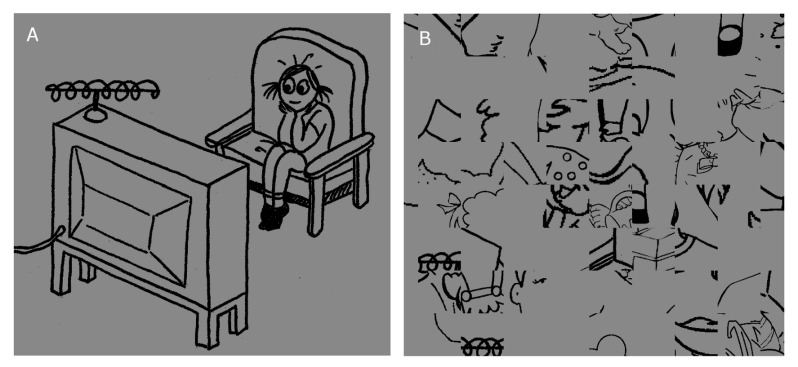
Examples of stimuli used in sentence generation and list generation tasks. **A**: An example of the line-drawing stimuli presented in the sentence generation tasks. **B**: An example of the scrambled line-drawing stimuli presented in the list generation task.

Mazoyer and colleagues (2014) conducted the largest study to date comparing language lateralisation with fMRI in left- versus right-handers. They used a sentence generation task in which on each trial the participant viewed a pictured scene (see Figure 1a for example) and had to generate a sentence to describe it. Participants were trained to produce a particular sentence type beginning with a subject (e.g. “the children”), followed by a description of the subject (e.g. “wearing aprons”), a verb (e.g. “cook”) and ending with a detail about the action (e.g. “in the kitchen”). As a comparison task, participants were asked to recite an overlearned word sequence (months of the year), while viewing a scrambled picture (see Figure 1b). Both the sentence generation task and the comparison task involved viewing a visual stimulus while generating words, so after subtracting the activation associated with the latter task from the former, the activation would reflect the additional brain activity elicited by selecting lexical items and assembling them into a grammatical sentence.

As will be discussed further below, because fTCD provides centisecond resolution for left-right blood flow differences, and the time course differs between tasks, it is not straightforward to simply subtract activation from a comparison task. Nevertheless, the potential benefits of having a comparison task to give a purer measure of activation during language generation are such that we decided to explore how feasible it would be to use a method in fTCD that was closely based on the approach of Mazoyer and colleagues. We predict that the effect of subtracting a comparison task would be the same as is observed in fMRI; i.e., it will yield higher LI values.

Our specific hypotheses were pre-registered on Open Science Framework (OSF) as follows:

Hypothesis 1: Individuals will be consistent in measures of hemispheric lateralization across different language domains; i.e., measures of lateralization (Lateralization index) will be statistically equivalent across word generation and sentence generation tasks.

Hypothesis 2: Lateralization indices acquired during the sentence generation task will be significantly larger when calculated relative to an active comparison task (number counting, i.e. automatic speech production) than when calculated relative to a resting baseline.

## Methods

### Deviation from pre-registered analysis plan

The analysis plan was pre-registered on OSF. There were three deviations from the pre-registered plan.

First, we have reworded hypothesis 2 to refer to an ‘active comparison task’ rather than an ‘active baseline task’, to avoid the confusion of ‘baseline’ being used with different meanings in fMRI and fTCD contexts (see above).

Second, data were analysed using custom scripts in R version 3.4.4 ( R Core Team, 2017) rather than using the publicly-available software package, DopOSCCI, developed by Badcock *et al.* (2012a) in Matlab. There were two reasons for this change: first, the R script had been developed in our group to fulfil the need for a reproducible and efficient method for processing large numbers of datasets, without using commercial (Matlab) software that required a licence (see Wilson & Bishop, 2018). As with DopOSCCI the analytic pipeline closely followed procedures developed by Deppe *et al*. (2004), with one additional option: the possibility of identifying brief periods of signal spiking or dropout and interpolating over these, to avoid rejecting trials. Wilson & Bishop (2018) compared results from DopOSCCI and the R script and found only small differences in the LIs computed by the two methods.

Third, 31 participants were tested instead of the planned group size of 30. The extra participant was recruited in case a dataset had to be excluded due to poor data quality, but ultimately all datasets were able to be used.

### Participants

31 participants were recruited using flyers and advertisements distributed throughout the University of Oxford (11 males, age range 17–42, mean age 25 years). As our focus was normal range variation in language laterality, we did not use handedness as a selection criterion, but we asked participants to self-report their preferred hand for writing (left, right or ambidextrous): the sample included two left-handers. Participants were recruited after screening to confirm they had no known speech, language or learning impairment, or other diagnosed neurological disorder. In addition to these 31 participants, there were 4 participants who were recruited but for whom no data was collected: insonation of the MCA was not possible in two participants because a suitable temporal window could not be found; one participant became faint during the fTCD set-up and data collection was aborted; one participant disclosed a developmental reading delay after screening, which made them ineligible for participation.

### Consent

Written informed consent for publication of anonymised data was obtained from the participants.

### Power analysis

The required sample size to detect lateralisation greater than zero was calculated using a power analysis based upon effect sizes reported from previous studies using fTCD in comparable participant groups. A meta-analysis of 12 studies ( Badcock *et al.*, 2012a; Bruckert, 2016; Dräger & Knecht, 2002; Grabitz *et al.*, 2016; Gutierrez-Sigut *et al.*, 2015; Illingworth & Bishop, 2009; Knecht *et al.*, 1998a; Krach & Hartje, 2006; Lust *et al.*, 2011; Somers *et al.*, 2011; Stroobant *et al.*, 2009; Whitehouse & Bishop, 2008) suggested that a sample size of 9 participants would be sufficient to detect the average effect size as found using the word generation task. However, as the expected strength of laterality with the sentence generation task is unknown, we planned to collect data from as many participants as possible within the time and resource constraints of the research team, with a goal of selecting a minimum of 30 participants.

### Task design

**Figure 2.**
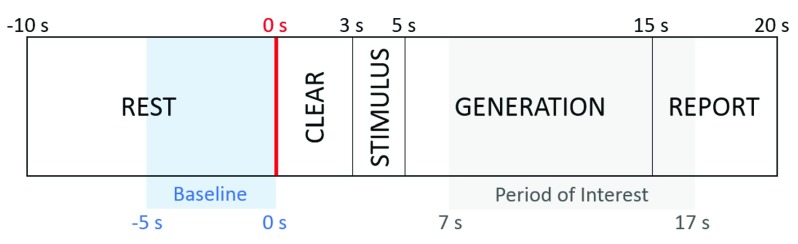
Schematic depicting the common structure of each trial. Schematic illustrating the timings used in each trial. The red line depicts the point at which the stimulus appears, while the period of interest is shown in grey, from 7 to 17 seconds. The blue area depicts the 5 second resting baseline period.

All participants performed three tasks: the ‘gold-standard’ word generation (WG) task, sentence generation (SG) and list generation (LG). The task script and stimuli can be found at OSF ( Woodhead *et al.*, 2018). The trials were presented using Psychophysics Toolbox version 3.0.13 ( Brainard, 1997; Kleiner *et al.*, 2007) in MATLAB 2012b software (Mathworks Inc.), with 20 trials each of word, sentence and list generation interleaved in a pre-determined pseudorandomised order. Each trial had a common structure, as depicted in Figure 2. Participants were first asked to clear their mind, before the presentation of the task-specific stimulus for 2 seconds. This was followed by the presentation of a black cross in the centre of the screen for a duration of 10 seconds, indicating that participants should covertly generate the speech required for that trial type. After this period of covert generation, participants were prompted to “Report”, and overtly state any speech they generated in the previous covert period. A period of covert speech generation allows measurement of changes in blood flow without the presence of any motor artefacts from speech production. Participants were then instructed to rest for 10 seconds. The rationale for the use of this particular structure was based upon a study by Gutierrez-Sigut and colleagues (2015), in which this same trial structure revealed strong effect sizes across both phonological and semantic fluency tasks, with a period of covert generation before overt report. Covert generation was shown to result in higher quality data in that fewer epochs were rejected from analysis.

*Word generation task (WG).* Following the structure presented in Figure 2, a single letter was presented as the stimulus on each trial of this task, indicating to the participant that they should generate as many words as possible beginning with that letter.

*Sentence generation task (SG).* The design was based on Mazoyer and colleagues (2014), though different stimulus materials were used to be more culturally appropriate for the UK. Participants were presented with a simple black line drawing on a grey background as the stimulus for this task. They were instructed to generate a sentence of a particular structure based on the image presented: beginning with a subject (e.g. “the children”), followed by a description of the subject (e.g. “wearing aprons”), a verb (e.g. “cook”) and ending with a detail about the action (e.g. “in the kitchen”). Training before the task ensured that participants were comfortable with this sentence structure. An example of the line-drawing stimuli is depicted in Figure 1a.

*List generation task (LG).* LG acted as a comparison task, in which participants were instructed to repeatedly recite an overlearned word sequence, i.e., numbers one to ten. We picked this task after piloting showed that reciting months of the year (as used by Mazoyer *et al.*, 2014) was too demanding for many participants and led to poor behavioural performance. On list generation trials, participants were presented with a scrambled black line-drawing on the same grey background, to control for any possible perceptual effects, as depicted in Figure 1b.

### Apparatus

fTCD data were acquired using a Doppler ultrasonography device (Doppler-Box ^TM^ X) with bilateral probes held in place using a DiaMon headset (DWL Elektronishe Systeme, Singen, Germany). Stimuli were presented on a desktop computer.

### Procedure

After familiarisation with the stimuli with brief training exercises, the velocity of the blood flow in the left and right MCA was measured and recorded. A suitable, stable signal was isolated from each MCA before the start of the task, through the positioning of 2-MHz transducer probes, attached to a headset, bilaterally over each temporal window. The MCA was isolated at a depth of 45–65 mm with a velocity of 35–100cm/s, with its identity confirmed by increasing the signal depth until there was a bifurcation of the signal corresponding to the anterior communicating artery. Trials were split into two separate runs, each containing 30 trials, with a short break in between runs. Behavioural performance was recorded and the number of words spoken in each condition was noted. Trials in which participants failed to respond, or where the signal was lost from the probe, were marked to be excluded from analysis – this affected 4.65% of all trials.

### fTCD Analysis

A custom R script (WSLG_Analysis.R, available from OSF ( Bishop *et al.*, 2018b)) was used to obtain a laterality index (LI) from the raw cerebral blood flow velocity (CBFV) data from left and right channels.

The following steps are involved in data processing. Raw data are first down-sampled from 100 Hz to 25 Hz. The data are then segmented into epochs of 30 seconds, beginning 5 seconds before the presentation of the trigger stimulus for each trial. Before further processing, the script displays data for each trial, enabling the user to identify cases where there is a brief spiking or loss of signal: the signal was interpolated if the signal had an extreme value (quantile value < .0001 or >.9999) affecting just one time point; trials with longer periods of signal loss were excluded.

The next step is normalisation such that the values for blood flow velocity become independent from the angle of insonation (the angle at which the Doppler probe observes the flow of blood through the MCA) and the diameter of the MCA. Heart cycle integration then removes rhythmic modulations in blood flow velocity. Each epoch was baseline corrected using the baseline interval set within the 5-second rest period before trigger presentation for the resting baseline condition ( Figure 2). Artefacts were identified as values below 60% and above 140% of mean CBFV and excluded from later analysis.

For each task, the left hemisphere CBFV minus right hemisphere CBFV is computed (L-R difference wave), and the point of maximal difference (the peak) within a predefined period of interest is identified. The period of interest began four seconds after stimulus presentation, and continued for 10 seconds, ending two seconds into the overt report period (see Figure 2). This is compatible with the time-course of the change in CBF in response to an external stimulus, which is thought to peak around 2–3 seconds after stimulus presentation ( Gagnon *et al.*, 2015; Payne, 2018). The Lateralisation Index (LI) was calculated by taking the mean of the L-R difference wave in a two-second time window centred at that peak.

The 95% confidence interval of the LI from individual trials was used to classify participants as left-lateralised if their average LI was significantly greater than zero, and as right-lateralised if the LI was significantly less than zero. Participants were classified as bilateral if confidence interval of the LI spanned zero.

### Statistical analysis

Statistical analyses were also performed in R version 3.4.4 ( R Core Team, 2017). Scripts are available from OSF ( Bishop *et al.*, 2018b). One sample t-tests were used to investigate whether the LIs from each task were significantly different from zero, and matched two-sample t-tests assessed whether significant differences were found between tasks in order to test the hypothesis that individuals are consistent in measures of lateralization across different language domains. To confirm reliability of our measurements across all three tasks, Spearman’s rank split-half correlations were calculated between odd and even trials. We also use Spearman correlations to explore the agreement between LIs for the three tasks. We had not planned any analyses of the effect of handedness, as we had only two left-handers in the sample, but we distinguish the left-handers visually in scatterplots.

Bland-Altman analysis ( Altman & Bland, 1983) was used to assess the degree of agreement between the gold standard word generation task and the sentence generation task and, in doing so, test the hypothesis that laterality indices obtained from these two tasks were statistically equivalent. This method considers whether agreement between two measures is within the limits that might be expected on the basis of knowledge of reliability of the measures. In effect, it helps establish whether the correlation between two measures is compatible with the correlation of each measure with itself (i.e., its reliability). There is no *a priori* rationale for determining the largest limit of agreement that is acceptable ( Giavarina, 2015), and so we based this on pre-existing data from previous studies by our group for comparing odd and even trials for the Word Generation Task in a large sample of children. For this sample, the mean difference between odd and even epochs was 0.16 with standard deviation of 1.24, which gives limits of agreement from -2.27 to 2.61. For the current study, we specified a limit of agreement from -2.5 to 2.5: if values on the Bland-Altman plot fall within these bounds, then the two tasks are deemed to be equivalent.

A second laterality index from the sentence generation task (SG2) was also computed using list generation as the comparison task. As noted above, there are various ways this could be done. The simplest approach is to compute the laterality index for each task separately, and then subtract the list generation LI from the sentence generation LI. Our pre-registered analysis specified we would conduct a one-tailed t-test with alpha level set to 0.025 to test the directional prediction that LI values from sentence generation with an active baseline will be larger than those with a resting baseline. Given that the LI is computed from the peak value, this may involve comparing LIs from different time windows. In additional exploratory analyses, we considered whether alternative methods might be preferable, to avoid this problem. One approach is to subtract the L-R difference wave obtained for the list generation task from the difference wave for the sentence generation task, and then compute a laterality index as usual, based on the peak value of this difference of differences waveform. A final approach, which might appear more compatible with fMRI, is to take the mean value of the difference wave within the period of interest for both SG and LG, and compute SG-LG. This approach does not require a peak to be identified.

In further exploratory analyses, we considered two questions: (a) whether the laterality index on a task was related to the number of words produced, and (b) whether bimodality of laterality indices was an artefact of the method of computation, which involved identifying a peak. To do this, we compared the standard method of LI calculation with an alternative approach that involved subtracting the mean activation over the period of interest for the right channel from the mean for the left channel. We then compared LIs for the two approaches, to consider how far they agreed, and whether the latter method gave a more normal distribution of LIs.

## Results

### Descriptives

**Figure 3.**
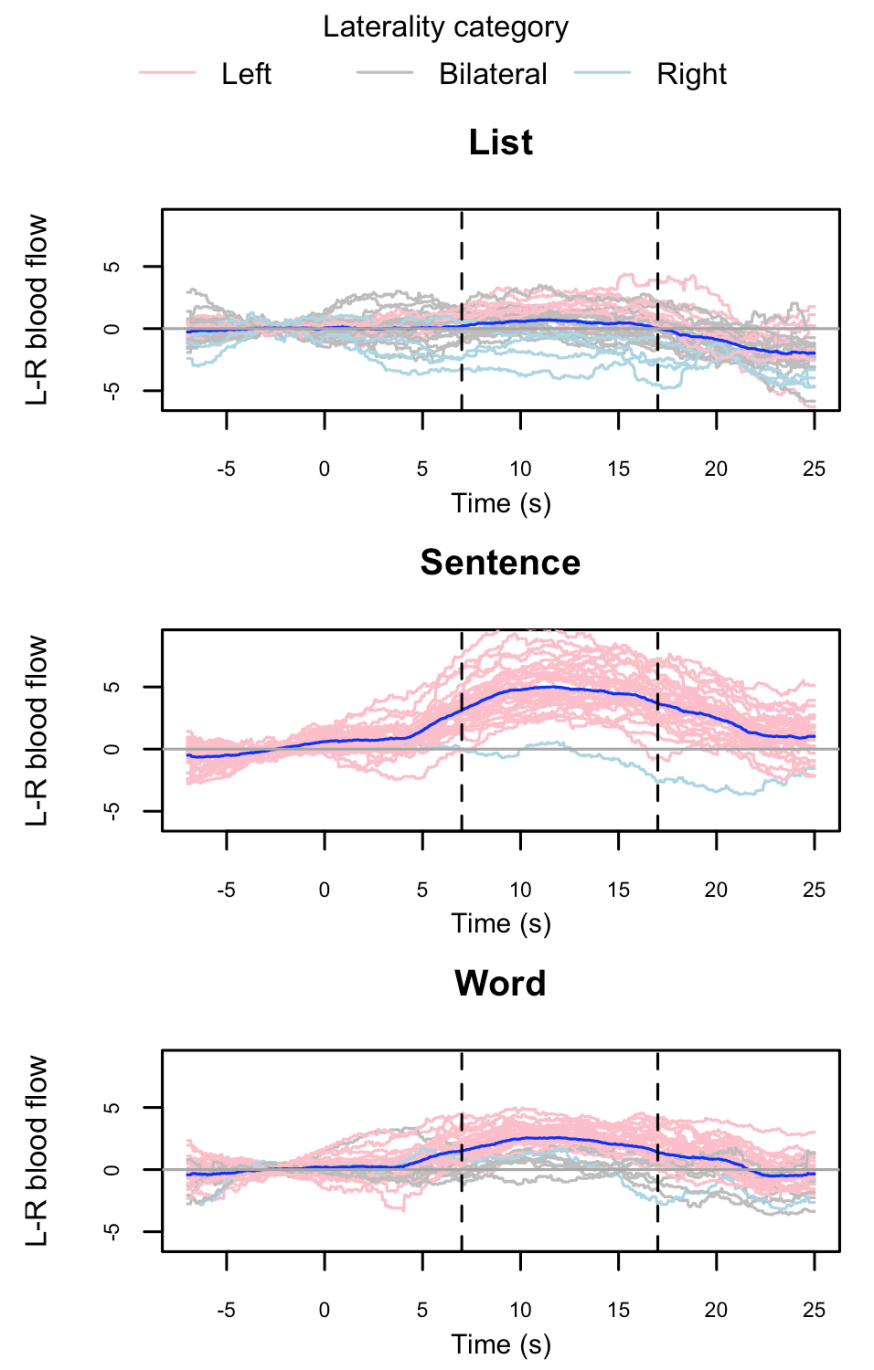
Time course of activation for each task. Bold line shows group mean, pale lines are individual participants, colour coded by laterality category for that task (red = left lateralised; grey = bilateral; blue = right lateralised).

**Figure 4.**
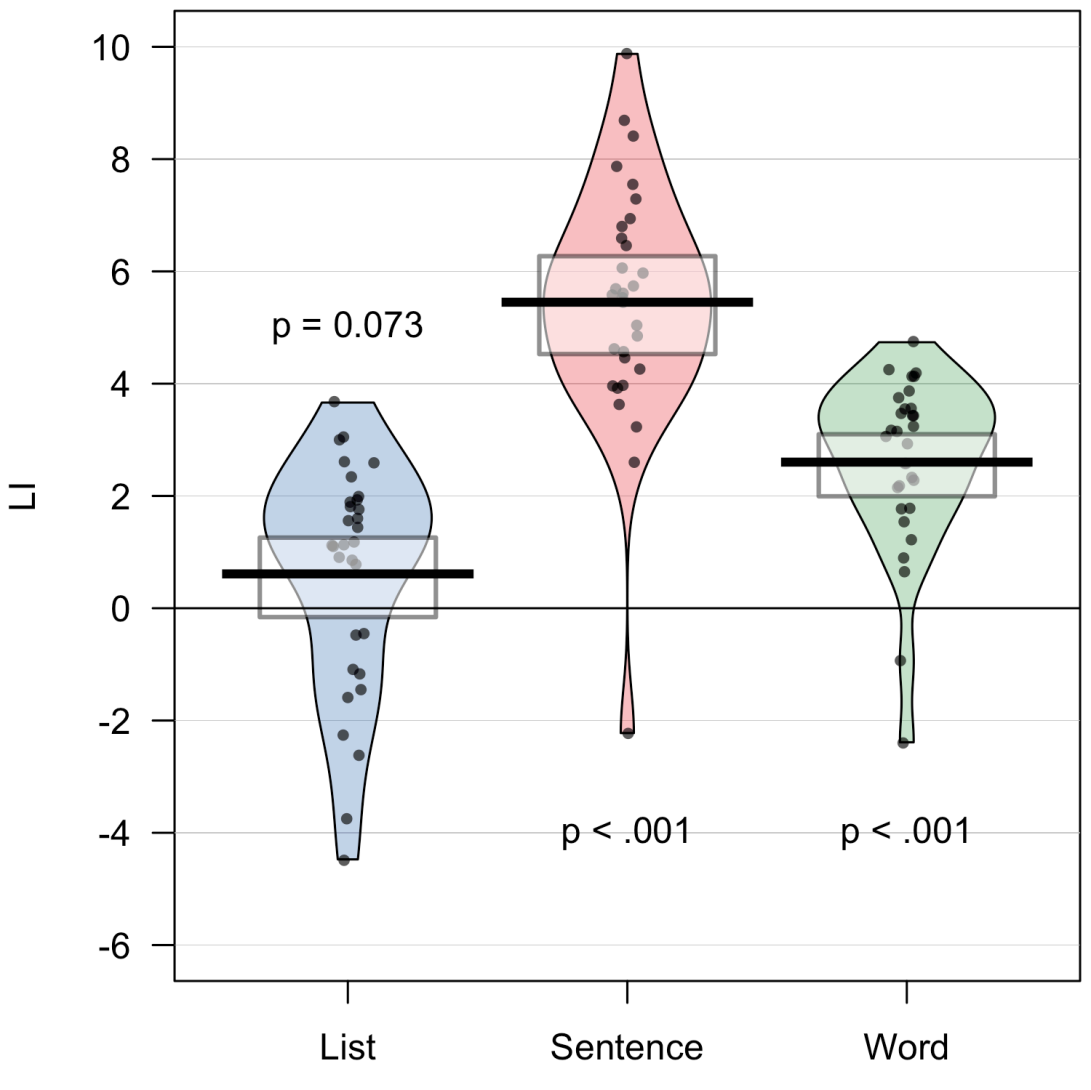
Pirate plot of lateralization index (LI) values. Pirate plot ( Phillips, 2017) showing distributions of individual LI values for each task, with the bold line indicating the mean. The results of 1-sample Wilcoxon tests comparing the LI values to zero (no lateralisation) are also reported.

The summary of key results from each task is found in Table 1. Shapiro-Wilk tests confirmed that LI values from the three conditions were not normally distributed (LG: p = .024; SG: p = .030; WG: p = .004). Spearman’s rho split-half correlations between odd and even trials from each task provide an index of the reliability of the LI values. The time-course of activation for each task is shown for each task in Figure 3. The distributions of LIs for each task are shown in Figure 4.

**Table 1.** Descriptive statistics of functional transcranial Doppler sonography (fTCD) data. Mean (SD) statistics for each task, including number of trials, mean N words produced per trial, lateralization index (LI) and peak latency, the number of participants that were left, bilateral or right lateralised, and Spearman’s rho for split-half (odd-even) reliability of the LI, with 95% bootstrapped CI.

	List	Sentence	Word
N trials	18.87 (1.36)	18.65 (1.20)	18.94 (1.12)
N words produced LI	10.12 (2.57) 0.61 (2.04)	9.13 (1.12) 5.45 (2.21)	4.34 (0.72) 2.60 (1.55)
Latency	12.47 (2.77)	12.65 (2.76)	11.61 (2.86)
N left	10	30	23
N bilateral	17	0	7
N right	4	1	1
Split-half r	0.73 (.49-.87)	0.82 (.60-.94)	0.46 (.10-.70)

The data in Figure 4 are so striking that statistics are hardly necessary. Results of one-sample Wilcoxon tests are shown alongside the data, confirming that whereas the WG and SG tasks are strongly lateralised, the LG task is not. Lateralisation in the SG task was substantially higher than for the WG task.

### Hypothesis 1: comparison of WG and SG tasks

**Figure 5.**
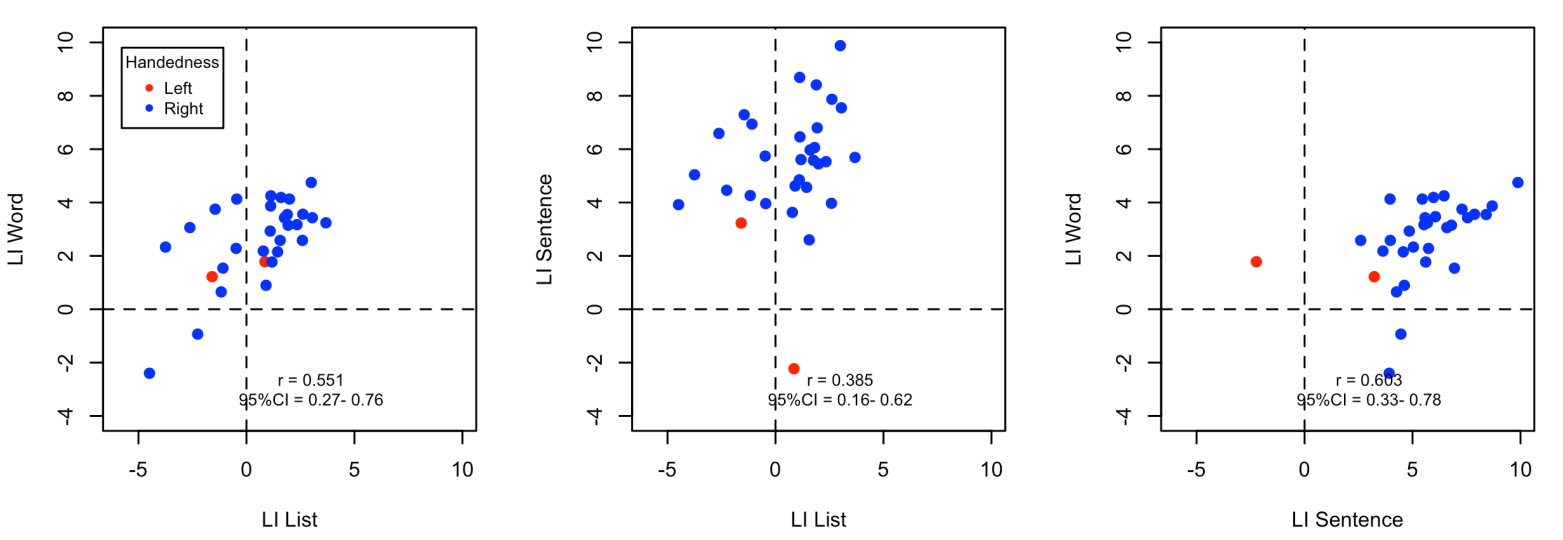
Pairwise scatterplots of lateralization index (LI) values. Scatterplots showing the pairwise comparisons of LI values between tasks. Spearman correlation coefficients (and 95% confidence intervals) are also reported. Right handed participants are indicated with blue dots; left handed participants with red dots.

All three tasks were significantly intercorrelated, as shown in Figure 5. Handedness is colour-coded, though we had too few left-handers to test for effects of handedness.

**Figure 6.**
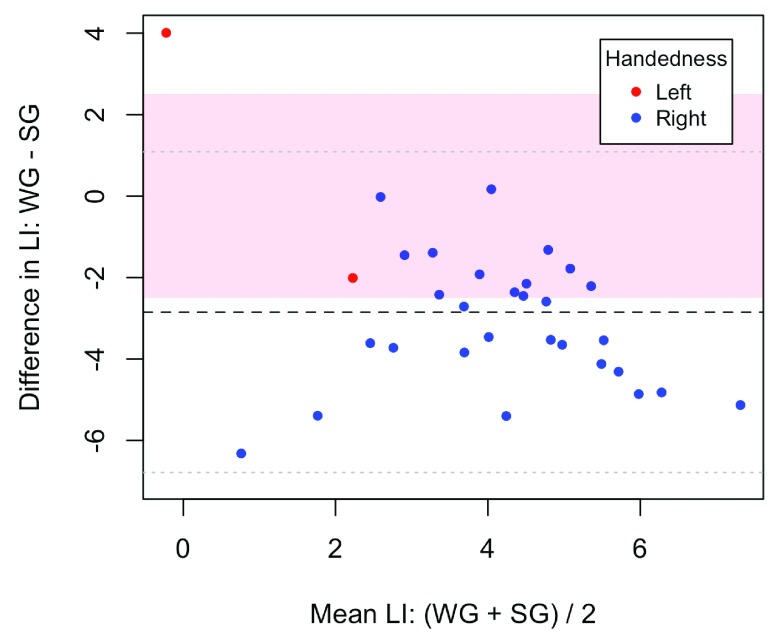
Bland-Altman plot comparing lateralization indices (LI’s) for Sentence Generation and Word Generation. The pink shaded region denotes the predetermined limits of agreement. The dashed line is the mean difference between Sentence and Word Generation LI values, and the dotted lines are the 95% confidence interval around the mean. Right handed participants are indicated with blue dots; left handed participants with red dots.

The mean LI values for the SG task were higher than those for the WG test (Mann-Whitney test, V = 471, p < .001). A Bland-Altman plot supported the conclusion that the tests were not interchangeable. As shown in Figure 6, the mean difference in LI values for the WG task versus the SG task (WG minus SG, dashed line) was outside of the predetermined limits of agreement (the pink shaded area). This is because LI values for SG tended to be higher than for WG.

### Hypothesis 2: Subtracting list generation from sentence generation LI

Our second hypothesis concerned the question of whether we would see stronger lateralisation on the sentence generation task if we followed the fMRI method adopted by Mazoyer *et al.* (2014) and subtracted the activation from list generation. Because this kind of subtraction is not customary in fTCD studies, there is no agreed procedure, and accordingly, we explored three different approaches:

A) Compute a LI as usual for SG and LG, and subtract the LG value from the SG value. This involves using the LI values already derived for the analyses presented above, and is the method we specified in our preregistration.
B) Subtract the L-R difference waveform for LG from the L-R difference waveform for SG, and identify the peak difference, with the LI corresponding to the mean blood flow around the peak, as done previously.
C) Subtract the mean L-R flow in the period of interest for LG from the mean L-R flow in the same period for SG.

**Figure 7.**
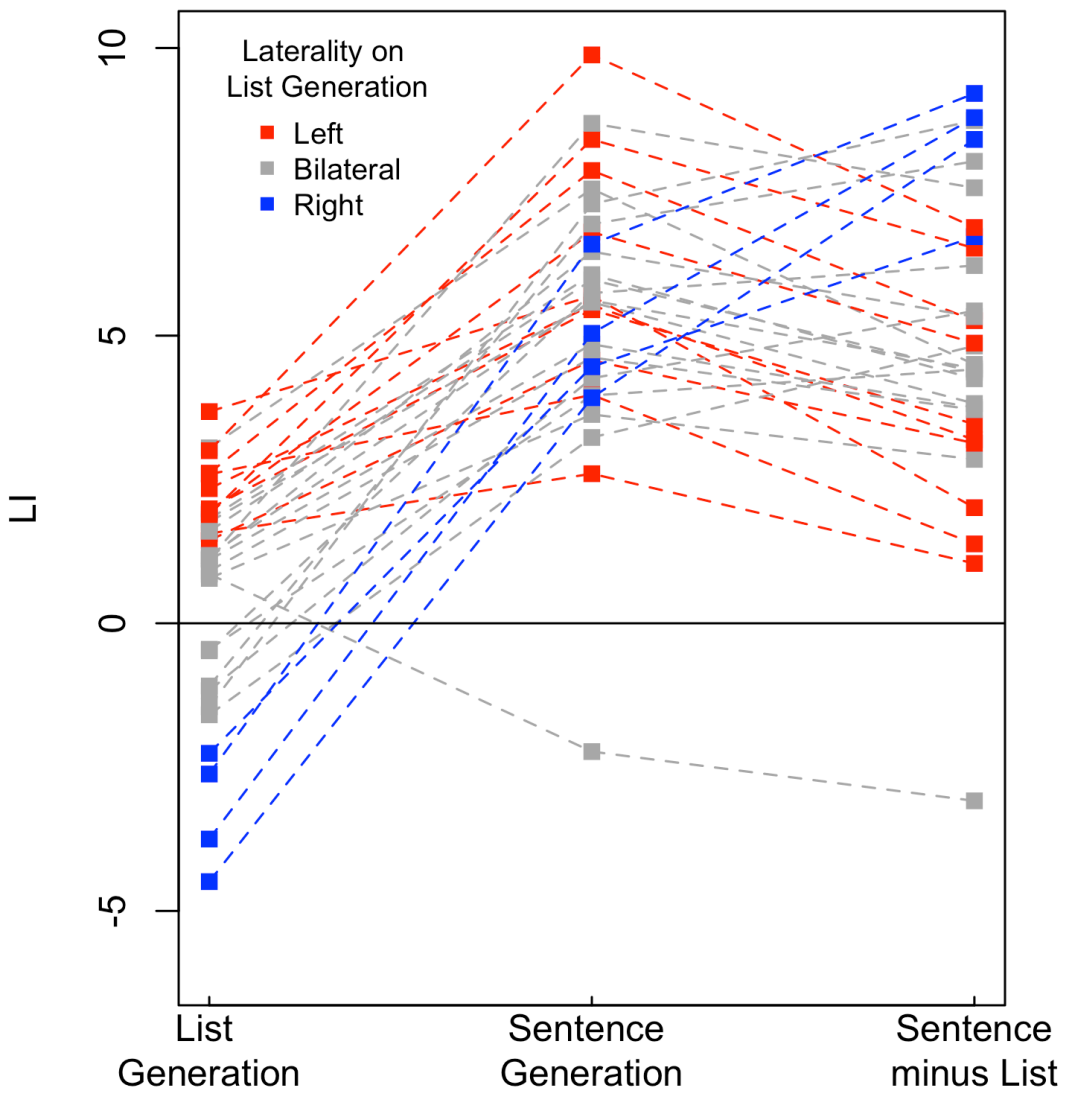
Effect of subtraction analysis on lateralization index (LI) values. Laterality indices for raw List Generation, raw Sentence Generation and the difference between Sentence Generation and List Generation for all individuals (using subtraction approach A).

The pre-registered paired comparison between the original LI for SG and the LI with approach A was not statistically significant (Mann-Whitney test: V = 340, p = .073). Results from the other methods of computation are simply summarised: results obtained with the three different subtraction methods were highly intercorrelated (all correlations greater than .90), and in no case did the use of a subtraction procedure increase the LI for sentence generation. The correlations for the original LI for SG and the LIs using the three subtraction methods ranged from 0.66 to 0.79. Nevertheless, although the group mean and SD were similar for LIs with and without the subtraction, the subtraction had a clear impact on the rank ordering of LIs. Figure 7 shows LI data from the LG and SG tasks, plus SG with subtraction method A. In effect, with the subtraction method, those who have very similar LIs for SG and LG will see reduced LI-A, those with bilateral LI on LG will not change, whereas those with right-biased LI for LG and left-biased LI for SG will become more left-biased on LI-A, because a negative number is subtracted from the raw LI.

### Exploratory analysis

***a) Does the strength of LI relate to the number of words produced in a task?***

A question that is often raised is whether language laterality depends on how challenging a task is. We previously failed to show any association of amount of verbal material produced and LI on verbal tasks, when manipulating difficulty within-subjects ( Badcock *et al.*, 2012b). In the current study, we observed higher LIs and more words produced in the Sentence Generation task than the Word Generation task, suggesting that verbal production may influence strength of lateralisation (see Table 1). However, the number of words produced in the List Generation task was greater still, and this task had much weaker lateralisation. We also considered the Spearman correlations between N words produced and LI within each task. None of the correlations reached significance: LG, r _s_ = -0.19, p = .30; SG, r _s_ = 0.10, p = .61; WG, r _s_ = -0.06, p = .73. These results indicate that the differences in LI observed between tasks was not simply due to behavioural differences in the amount of speech produced. However, it is worth noting that the LI values were calculated from fTCD recordings during the speech generation (planning) phase, rather than overt speech production; in contrast, Gutierrez-Sigut *et al.* (2015) noted a difference between overt and covert word generation in word fluency tasks. For phonological fluency, there was a significant relationship between the amount of speech and LI values during an overt condition, but not when covert speech was used.

***b) Is the bimodality of laterality indices an artefact of the method of calculation?***

**Figure 8.**
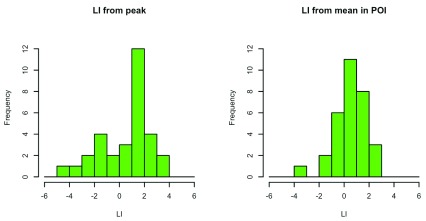
Impact of the method of calculating lateralization index (LI) on LI distribution. Distributions of LI values for list generation, computed from average around the peak, and mean over the period of interest.

The standard method of computing a LI with fTCD was developed by Deppe and colleagues (2004), and involves finding a peak in the difference wave and then computing the mean amplitude around that peak. This inevitably will induce bimodality in the distribution of LIs, as seen in Figure 4 for list generation, where a point of rarity occurs around zero. An alternative method of computing a laterality index was described above (method C) – taking the mean of the difference wave in the period of interest. Using this measure, LI values for the three conditions were as follows: for List Generation, LI = 0.48 (sd = 1.32); Sentence Generation LI = 4.58 (sd = 1.88); Word Generation, LI = 2.05 (sd = 1.17). Figure 8 shows the distribution of scores on the list generation task using this method compared with the traditional peak-based method. It is evident from inspection that the bimodality of the laterality index distribution is not seen when the means-based method is used. Despite the very different shapes of distributions from the two methods, they were highly intercorrelated: r _s_ = 0.94.

## Discussion

The aims of this study were to determine (a) whether a laterality index from a sentence generation task was equivalent to the gold-standard word generation task, and (b) whether subtracting a laterality index from a comparison list generation task would enhance the laterality index. We compared the lateralisation indices of a group of 31 participants across three tasks: word generation (WG), sentence generation (SG) and list generation (LG). Regarding the first hypothesis, we found that the WG and SG tasks were not equivalent, as laterality was significantly stronger for the SG task. The correlation between the two tasks was moderately strong (r _s_ = 0.60). These findings lend some tentative support to the idea that language lateralisation is not a unitary function, and that in some individuals, different components of language may be preferentially processed in different hemispheres. Further support for this idea comes from a previous fMRI study comparing lateralisation as measured by WG and SG in a clinical population, which revealed that masses in the left-hemisphere Wernicke’s area led to reduced lateralisation indices in this region for the sentence generation but not word generation, while the reverse was found for Broca’s area ( Partovi *et al.*, 2012). While this was a small study with task demands different to our own, it highlights a possible division of labour for the processing of different language components.

In fMRI studies, it is customary to subtract activation from a comparison task from activation associated with the language task of interest, with the aim of obtaining a purer measure that removes activation associated with more general motor or perceptual processing. To test our second hypothesis we considered whether we could achieve a similar effect by subtracting the LI from a list generation task from the sentence generation LI. The two tasks were well-matched in terms of the number of words spoken, but list generation did not show significant laterality at the group level. It was therefore not surprising that the subtraction had no effect on the laterality index for sentence generation at the group level.

An unanticipated finding, illustrated in Figure 5 and Figure 7, is that laterality on list generation was significantly correlated with laterality on the other two tasks. This was surprising: the overall lack of lateralisation at the group level might suggest that both hemispheres participate equally in this task, and any individual variation is not reliable or meaningful. The data, however, suggest that individuals do vary in the hemisphere used to generate over-learned lists, even though there is no bias to either side at the group level. The fact that the LIs from word and sentence generation tasks are correlated with list generation suggests that common processes of speech production are implicated in both tasks, with an additional left-sided bias superimposed that is specific to generation of novel, meaningful language.

This finding does not give a conclusive answer to the question of whether a list generation comparison task should be used in language laterality research: rather, it raises further questions. On the one hand, the logic of the comparison task subtraction methodology used in fMRI in this area is supported: any task that involves language generation will implicate speech production mechanisms, which do not appear to be lateralised at the population level. If we subtract this activation, we should obtain a clearer index of lateralisation specific to semantic and syntactic processing. Note, however, that, as shown in Figure 7, in doing this, we will allocate more extreme lateralisation indices to those who are inconsistently lateralised for list and sentence generation: this is an inevitable consequence of the algebra of the subtraction. This suggests an alternative interpretation of the findings of Mazoyer *et al*. (2014) regarding individuals with extreme atypical right-sided language laterality: these are likely to be those who are left-sided for list generation but right-sided for sentence generation. It may be that inconsistency between lateralisation of speech and language is more important than the direction or size of laterality of either alone.

In an exploratory analysis, we investigated whether the conventional method of computing the LI from the peak difference between left and right CBFV may produce a bimodal distribution of LI values. Figure 8 shows that such a bimodal distribution was observed for the list generation task, where LI values tended to zero. The alternative method of computing LI from the mean left minus right difference in CBFV within the period of interest has the advantage of producing normally distributed values, without a point of rarity at LI=0. Although the choice of the most optimal method for calculating LI may vary from task to task, we suggest that the latter method is a more veridical representation of CBFV asymmetry when task performance is sustained throughout the period of interest.

Our study was focussed on characteristics of typical language lateralisation, and a consequent limitation was that we had only two left-handed participants. The fact thatone of these left-handers was a clear outlier on the SG task may be of interest for future investigation. To explore this further, future work might investigate the relationship between tasks in a sample with more left-handers, using more quantitative assessments of handedness and language ability.

The technological limitations of the fTCD technique also cannot be overlooked – as mentioned above, poor spatial resolution means that functions can be localised only at a hemispheric level and the requirement of a suitable temporal window means a satisfactory signal cannot be obtained in around 5% of participants ( Lohmann *et al.*, 2006). Indeed, we had two such exclusions whereby suitable window could not be found, and one individual from which data could not be obtained as set-up was aborted.

## Conclusion

We adapted the sentence generation task of Mazoyer *et al*. (2014) for use with fTCD and demonstrated that it gives robust left-lateralisation in a group of unselected individuals, with good split-half reliability. The lateralisation on this task was significantly stronger than that seen on the gold-standard word generation task, although LIs from the two tasks were correlated. An unanticipated finding was that the LI from a list generation task, which was used as a comparison task for sentence generation, was significantly correlated with the LIs from both word generation and sentence generation, even though at the group level, this task was not lateralised. Subtraction of the list generation LI from sentence generation LI had no impact on the mean sentence generation LI, though it did affect the rank ordering of LI values. Those with extreme values on the subtracted Sentence-List LI will be those who have discrepant laterality across the two tasks.

In sum, we conclude that the sentence generation task is a reliable and feasible task for use with fTCD, which may be useful for identifying individuals who are inconsistent in lateralisation across different language domains. There may be value also in further studies with list generation, which allows one to study laterality of speech production when there is no role for using semantic or syntactic processes to generate novel sequences. The lack of an overall lateral bias on list generation might suggest that both hemispheres participate equally in speech production, but our data indicate that there are meaningful individual differences in left- or right-sided bias on this task, whose functional significance remains to be established.

## Data availability

All raw data, analysis scripts and processed data can be found on Open Science Framework.

Data:

CANDICE A2a: Measurement of language laterality, https://doi.org/10.17605/OSF.IO/68KEZ ( Bishop *et al.*, 2018a)

This dataset is available under a CC0 1.0 Universal license.

Task scripts:

CANDICE A2a: Measurement of language laterality, https://doi.org/10.17605/OSF.IO/EYVDB ( Woodhead *et al.*, 2018)

This dataset is available under a CC0 1.0 Universal license.

R analysis scripts:

CANDICE A2a: Measurement of language laterality, https://doi.org/10.17605/OSF.IO/X5DWZ ( Bishop *et al.*, 2018b)

This dataset is available under a CC0 1.0 Universal license.
