# Supplementary material for: Measurement of language laterality using functional transcranial Doppler ultrasound: a comparison of different tasks
Source: Wellcome Open Res. 2020 Feb 11;3:104. Originally published 2018 Aug 24. [Version 3] doi: 10.12688/wellcomeopenres.14720.3 (PMC6171558; doi:10.12688/wellcomeopenres.14720.3)
Supplement: Peer review history

# Peer review: Reviewer response for version 3

**DOI:** 10.21956/wellcomeopenres.17247.r38111

**Published:** 2020-05-06

**Authors:** Andreas Jansen

**Affiliations:** 1 Department of Psychiatry and Psychotherapy, Philipps University of Marburg, Marburg, Germany

Woodhead *et al*. assessed language lateralization using functional Doppler sonography (fCTD). Language lateralization was described by a laterality index (LI). They investigated the impact of different language tasks as well as the impact of different calculation methods on the LI.

 This is an interesting study, assessing important methodological aspect of fCTD. The study has several strengths, among others the preregistered analyses and the disclosure of all analysis methods. It is quite interesting to see that the Deppe *et al.* (1997) approach (summarized in Deppe *et al.* (2004)) tends to overestimate (the absolute value) of the LI.

 In my opinion however, some aspects of the study are poorly described. I will try to explain my impression in the following.

 The **abstract** is poorly written. Many statements are too unspecific. It is difficult for the reader to understand the aim, the results and the conclusions based on the information from the abstract alone. To give some examples:

Example 1: “Language laterality has mostly been studied with fTCD using a word generation task, but it is not clear whether this is optimal…” and this was a pre-registered methodological study designed to explore novel approaches to optimising measurement of language lateralisation using fTCD…”. What does “optimal” mean in this context? A task that is as short as possible? A task that yields more reliable LIs than previous tasks? A task that yields LIs that are more strongly lateralized? A task has a higher agreement with other technique, e.g. fMRI or the Wada test?

Example 2: “Bland-Altman analysis showed that these two methods were not equivalent.” It is impossible to understand what that means when reading only the abstract. The authors might want to describe their hypotheses in more detail also in the abstract.

Example 3: “The comparison list generation task was not systematically lateralised, but nevertheless laterality indices (LIs) from this task were significantly correlated with the other two tasks.” Again, when reading only the abstract the reader does not get useful information.

 Since I am myself not a native English speaker I will refrain from any specific advice how to improve the abstract. I suggest however that the authors rewrite the abstract so that is is understandable on its own.

 I also suggest that the authors rewrite some parts of the **background**. First, some paragraphs make statements that are rather vague. Second, some information given are too uncritical with regard to the properties of fTCD.

 To give one example for unspecific statements: “It is well-established that language is predominantly processed in the left cerebral hemisphere in the majority of people. The functional significance of this bias is not understood.” What is meant by the second sentence? It is a statement that is so unspecific that it is almost meaningless.

 To give some examples for an uncritical description of the fCTD literature: The authors state that “the word generation task (Knecht *et al*., 1998a; Knecht *et al*., 1998b) has been used as the gold standard method for assessing cerebral lateralisation using fTCD – with good concordance with fMRI (Deppe *et al*., 2000; Somers *et al*., 2011) and Wada technique (Knecht *et al*., 1998a; Wada & Rasmussen, 1960), and adequate test-retest reliability (Knecht *et al*., 1998a).”

 The literature cited, in particular the early fTCD studies, have several methodological shortcomings. To give two examples from the studies that are mentioned:

 Deppe *et al.* (2000) calculated LIs for a word generation task using both fMRI and fTCD. Aim of the authors was to assess the concordance of both methods, not only with regard to the hemispheric dominance (left/right/bilateral), but also with regard to the degree of lateralization. They reported a correlation of r=0.95 between both techniques. If you take a closer look at this publication, you will see that there remain open questions. How was the LI for fMRI data calculated? What statistical threshold was used? Why is the correlation between both techniques so high although the authors chose a calculation method for the LI-fMRI that has been shown to be not reliable (with regard to the degree of lateralization) when subjects are measured twice with fMRI? In my opinion, the results of this study do not fit together with many other studies, so its validity must be doubted. If the authors disagree, they might want to explain why the correlation between fTCD and fMRT is so much higher than for repeated fMRT measurements.

 Knecht *et al.* (1998) compared fTCD with the Wada test. They concluded that their study “validates fTCD as a noninvasive and practical tool for the determination of language lateralization that can be applied for clinical and investigative purposes.” When you want to apply fTCD for clinical purposes however you have to be able to assess whether a subject shows typical or atypical language dominance. Atypical language dominance includes both bilateral as well as right-hemispheric dominance. Why did Knecht and colleagues not include this category in their analysis? Is the correlation reported (r=0.92) also that high when only including subjects with typical language lateralization?

 I cannot go into detail within the scope of this review. But there are many reasons why in particular the early fTCD publications overestimate the reliability of the technique and its concordance with other imaging modalities dramatically. I therefore think that statements such as “good concordance with fMRI” or “adequate test-retest reliability” should be used with caution.

 The – preregistered - **hypotheses** that are stated at the end of the introduction are with regard to their verbal formulation is not sufficiently specific. What is for instance meant by “individuals will be consistent in measures of hemispheric lateralization across different language domains”. Do you expect that if a subject is left-dominant for one task she is also left-dominant for another task? Or do you expect that a subject that is more strongly left-lateralized than another subject as assessed by task 1 is also more strongly left-lateralized when using task 2? These are different scenarios!

 To give another example: Do you intend to test hypothesis 2 by comparing the mean LI (averaged across all subjects) between both implementations or on a single subject level? The authors might try to specify their hypotheses more clearly on a verbal level. Alternatively, they should describe more clearly in the methods section which analysis is used to test which hypothesis. At present, it takes some time for the reader to clearly disentangle which analysis is used for which reason. To me, it did not become fully clear. In my personal opinion, the manuscript would benefit from a more structured presentation of the analyses.

 The **power analysis** calculated the required sample size to detect lateralisation greater than zero. Can it be that this power analysis is no way related to the specific questions stated in the introduction?

**Descriptives**: Do the authors expect that the LIs are normally distributed? Typically, you expect a bimodal distribution (see e.g. Knecht 2000), some are left-dominant, some are right-dominant. When considered separately, the LIs for subjects with left- and right-dominance might be normally distributed. Does it make sense to test for normality for the total sample?

Is the work clearly and accurately presented and does it cite the current literature?

Partly

If applicable, is the statistical analysis and its interpretation appropriate?

Yes

Are all the source data underlying the results available to ensure full reproducibility?

Yes

Is the study design appropriate and is the work technically sound?

Yes

Are the conclusions drawn adequately supported by the results?

Yes

Are sufficient details of methods and analysis provided to allow replication by others?

Yes

Reviewer Expertise:

Cognitive neuroscience, functional neuroimaging.

I confirm that I have read this submission and believe that I have an appropriate level of expertise to confirm that it is of an acceptable scientific standard, however I have significant reservations, as outlined above.

# Peer review: Reviewer response for version 3

**DOI:** 10.21956/wellcomeopenres.17247.r38110

**Published:** 2020-03-16

**Authors:** Guy Vingerhoets

**Affiliations:** 1 Department of Experimental Psychology, Faculty of Psychology and Educational Sciences, Ghent University, Ghent, Belgium

The study aims to contribute to two important questions in laterality research: is sentence generation equivalent to word generation when it comes to the determination of hemispheric asymmetry of language laterality and is task subtraction used in fMRI also applicable in fTCD?

 This clearly written paper has been commented on by two reviewers already, and their suggestions have been included in adapted versions of the paper. In addition, a problem with the original analysis has been corrected. The amendment resulted in a significantly stronger laterality index for the sentence generation task than for the word generation task.

 Given all this previous work fine-tuning the paper, it appears to me that the available data have been analyzed thoroughly and that the conclusions are supported by the results obtained.

 So, task subtraction does not enhance the laterality index in fTCD and sentence generation produces stronger laterality indices than word generation.

 The former finding does not lead to a specific recommendation though, as subtraction might have advantages as well as disadvantages.

 With regard to the latter finding, I was wondering whether this finding might influence the authors’ opinion with regard to the status of word generation as the gold-standard for language laterality.

 In the abstract the authors mention that it is unclear whether the use of a word generation task in fTCD is optimal for measuring language laterality. What is meant by optimal? If a stronger laterality index is better, should we then change the gold-standard to sentence generation? Or should we only acknowledge that different language tasks have different LI’s but agree to keep on using word generation as the prototypical language task giving its widespread use and validation?

 In sum, I believe this manuscript provides valuable information that contributes to our knowledge on the measurement of language laterality and can be approved for indexing.

Is the work clearly and accurately presented and does it cite the current literature?

Yes

If applicable, is the statistical analysis and its interpretation appropriate?

Yes

Are all the source data underlying the results available to ensure full reproducibility?

Yes

Is the study design appropriate and is the work technically sound?

Yes

Are the conclusions drawn adequately supported by the results?

Yes

Are sufficient details of methods and analysis provided to allow replication by others?

Yes

Reviewer Expertise:

Neuropsychology, Brain asymmetry, Laterality, Hemispheric functional segregation, Brain organization.

I confirm that I have read this submission and believe that I have an appropriate level of expertise to confirm that it is of an acceptable scientific standard.

# Peer review: Reviewer response for version 1

**DOI:** 10.21956/wellcomeopenres.16036.r33764

**Published:** 2018-10-03

**Authors:** Eva Gutierrez-Sigut

**Affiliations:** 1 University of Valencia , Valencia, Spain

This is a clearly written and needed study that aims to optimise the measurement of Language lateralisation using fTCD.

 The study assess lateralisation indices of 31 participants during 3 different covert language production tasks, including the gold standard task (word generation). Results show that although similar and highly correlated, word generation and generation of sentences from pictures don't seem to be interchangeable tasks.  More interestingly, the pattern of results that arises when "list repetition" is used as comparison task suggests that word and sentence generation add a directional bias to a continuum of laterality. Intraindividual consistency in lateralisation between different different language tasks might be explanatory of how language processing occurs in the brain,

 I think that the authors efforts to constantly improve the pipeline of data pre-processing is commendable. Specifically the addition of an extra interpolation step which has the potential to result in lower number of valid trials rejected. I am curious of why the interpolations is performed after and not before down-sampling (which should theoretically provide better interpolation).

 The statistical analyses presented, the pre-registered and the exploratory, are clearly motivated and advance the field. Can the authors offer any recommendation on best practice for future studies based on these comparisons? e.g. is it better to avoid the bimodality in LI distributions?

 Although I can understand where hypothesis 2 comes from it could be more motivated in the intro. On the same note, the focus on the Mazoyer et al. (2014) ^1^ results is put on how the strongly right lateralised individuals were left handed. I am unsure of how this ties up with the present study. The reasoning might need a bit of unpacking.

 It is stated that the word and sentence generation tasks are not interchangeable, this is based on a very small percentage of participants (6 including the only two left handed participants) falling on the non-acceptable range for the Bland-Altman plot. Are the results from the two tasks really that different? If they are, what do the authors think that might be the difference? grammatical/syntactic processing that tends to be more frontal? task engagement? I think the authors propose a really interesting framework for future fTCD research and further discussion on what particular aspects of the tasks could affect LIs would be really valuable.

 The correlations between behaviour and LI are not significant, however this is not strange taking into account that the behaviour inputed to the correlation is for the report phase and LI is being measured before. 

 Very minor point: 

 Although the authors say the they have substituted the term "baseline task" by "comparison task" to avoid confusion I think baseline is used to refer to task further down the manuscript (see below). This might be intentional.

 "...LI values from sentence generation with an active baseline will be larger than those with a resting baseline..."

 "...This finding does not give a conclusive answer to the question of whether a list generation baseline should be used in language laterality research: rather, it raises further questions...."

Is the work clearly and accurately presented and does it cite the current literature?

Yes

If applicable, is the statistical analysis and its interpretation appropriate?

Yes

Are all the source data underlying the results available to ensure full reproducibility?

Yes

Is the study design appropriate and is the work technically sound?

Yes

Are the conclusions drawn adequately supported by the results?

Yes

Are sufficient details of methods and analysis provided to allow replication by others?

Yes

Reviewer Expertise:

Cognitive neuroscience; Deafness; Lateralisation; ERPs; Reading; Reading Difficulties

I confirm that I have read this submission and believe that I have an appropriate level of expertise to confirm that it is of an acceptable scientific standard.

## References

[1] : Gaussian mixture modeling of hemispheric lateralization for language in a large sample of healthy individuals balanced for handedness. *PLoS One*.2014;9(6) : 10.1371/journal.pone.0101165 e101165 10.1371/journal.pone.0101165 24977417PMC4076312

# Response

**Published:** 2018-10-11

**Authors:** Zoe Woodhead

**Affiliations:** University of Oxford, UK

We would like to thank the reviewer for their thoughtful and constructive comments, which we have addressed in the revised version of the manuscript as discussed below.

**1. Interpolation of fTCD artefacts**

We observe that spike artefacts in our fTCD are very brief – they may only last one or two samples. This means that there are, inevitably, fewer artefacts in the downsampled data than the raw data. Hence, it makes sense to perform the interpolation of spike artefacts after downsampling.

**2. Recommendations for use of LIs in future research**

As we report in the paper, using the mean L-R difference within the POI as a measure of LI (the ‘mean-based’ LI) results in more normally distributed LI values, and avoid the bias away from LI=0, which is a problem with conventional peak-based LIs. If your subsequent analyses rely on the assumption of normality, then mean-based LIs are definitely a better option. We also believe that it is a better reflection of the actual lateralisation of brain activity, as it is calculated from CBFV throughout the period of interest. In some individuals, we have observed that peak-based LI values can be driven by a transient period of lateralised CBFV that is not a real reflection of their overall pattern of CBFV across the epoch.

The decision of which measure to use may depend on the task in question – some tasks that require a transient event that occurs at a variable timepoint within the period of interest may be better suited to a peak-based method – but in general, we have started to use a mean-based measure as standard in our research.

We have updated the discussion to make this recommendation clearer to the reader.

**3. Motivation for hypothesis**

We have reorganised the Background section to highlight the key points from Mazoyer’s paper (Mazoyer et al., 2014), and have deleted some extraneous detail about this study. We hope that this makes the rationale behind Hypothesis 2 easier to understand.

**4. Interchangeability of word and sentence generation tasks**

We agree with the reviewer that it is hard to interpret the results from the Bland-Altman plot: the data do not support a conclusive interpretation. The LI values from the two tasks are not significantly different, but equally the Bland-Altman task (which was defined in our preregistration as the method we would use to assess similarity) identified some participants for whom the LI values differed between the two tasks. The data suggest that for most people, word and sentence generation have very similar levels of lateralisation, but that this isn’t the case for everyone. This may reflect meaningful heterogeneity in the population of typically developed adults in the organisation of language functions across the two hemispheres. The current data do not allow us to speculate further on this matter, but a more recent study we have conducted using a range of six different language tasks (Woodhead et al, 2018) suggests that this heterogeneity does exist, and (in line with Mazoyer’s findings) that an atypical pattern of language lateralisation can occur in a minority of participants. 

**5. Correlations with task performance**

We agree the amount of speech produced is less likely to be correlated with LI when it is recorded during covert speech than when overt speech is used (as was observed in Gutierrez-Sigut et al., 2015). Our main purpose in reporting these statistics is because there was a large difference between conditions in the amount of speech produced. We therefore wished to note that this behavioural difference was unlikely to have driven the results we saw between tasks in LI values. We have clarified this motivation in the Results section. Inclusion of correlations within individuals was just included for completeness: we suspected that if we did not report this, readers may have been curious about it. We agree it is not surprising to find no significant associations.

**6. Use of ‘baseline task’ or ‘comparison task’**

Thank you for pointing out this inconsistency! We have rectified the error and made sure that ‘comparison task’ is now used throughout.

**References**

Gutierrez-Sigut, E., Payne, H., & MacSweeney, M., Investigating language lateralization during phonological and semantic fluency tasks using functional transcranial Doppler sonography, *Laterality: Asymmetries of Brain, Body and Cognition*, **20**, 49-68 (2015).

Mazoyer B., Zago, L., Jobard, G., Crivello, F., Joloit, M.m Perchey, G., Mellet, E., Petit, L., Tzourio-Mazoyer, N., Gaussian Mixture Modeling of hemispheric lateralization for language in a large sample of healthy individuals balanced for handedness. *PLOS One*, **9** (2014).

Woodhead ZVJ, Bradshaw AR, Wilson AC, Thompson PA, Bishop DVM., Testing the unitary theory of language lateralisation using functional transcranial Doppler sonography in adults. *bioRxiv,* **437939,** doi: https://doi.org/10.1101/437939, (2018).

# Peer review: Reviewer response for version 1

**DOI:** 10.21956/wellcomeopenres.16036.r33849

**Published:** 2018-09-21

**Authors:** Ervin Sejdić

**Affiliations:** 1 Department of Electrical and Computer Engineering, Swanson School of Engineering, University of Pittsburgh, Pittsburgh, PA, USA

- A different statistical test may be needed, unless the authors have confirmed that LI points adhere to assumptions of t-tests.
- Is it possible also to include mean values for observed blood flow velocities?
- While LI is a typical feature to understand lateralisation, have you considered other signal features? LI is an intuitive measure, but also a very crude measure.

Is the work clearly and accurately presented and does it cite the current literature?

Yes

If applicable, is the statistical analysis and its interpretation appropriate?

Yes

Are all the source data underlying the results available to ensure full reproducibility?

Yes

Is the study design appropriate and is the work technically sound?

Yes

Are the conclusions drawn adequately supported by the results?

Partly

Are sufficient details of methods and analysis provided to allow replication by others?

Yes

Reviewer Expertise:

NA

I confirm that I have read this submission and believe that I have an appropriate level of expertise to confirm that it is of an acceptable scientific standard.

# Response

**Published:** 2018-10-11

**Authors:** Zoe Woodhead

**Affiliations:** University of Oxford, UK

In response to the first point, we have reported Shapiro-Wilk normality tests for the LI data, which did not indicate that LI values from any of the three tasks were non-normally distributed. This may seem odd, given the bimodal distribution observed for the List Generation task in Figure 4. The Shapiro-Wilk test result for this condition was p=.084. Statistically, it is impossible to say for certain whether the data is truly normally distributed, or whether low power (due to the relatively small sample size) affected the Shapiro-Wilk result. But intuitively, the method of identifying LI values from the peak in L-R difference seems bound to result in a bimodal distribution if the ‘true’ mean lateralisation is close to zero; hence, we feel justified in recommending a change in the way the LI values are calculated, despite the apparent normality of the conventional LI values reported here.

 For the second point, we have now reported the mean LI values using the alternative ‘means-based’ method at the end of the Results section. They were as follows: for list generation, LI = 0.16 (sd = 1.83); sentence generation LI = 2.48 (sd = 1.70); word generation, LI = 1.91 (sd = 1.30).

 For the final point, we agree with the reviewer that there may be other features of interest in the fTCD signal that are not part of the conventional analysis methodology; for example, examining the timecourse of blood flow may be a fruitful avenue of investigation (e.g. Meyer et al., 2014). However, our aim in the current study was to examine the validity of current standard practise in fTCD studies, and the analyses we carried out to achieve this aim were planned in our preregistration. We would like to conduct further research on whether more novel methods may give additional insights into lateralisation, as the reviewer suggests.

**Reference**

 Meyer G., Spray A., Fairlie J., Uomini N., Inferring common cognitive mechanisms from brain blood-flow lateralization data: A new methodology for fTCD analysis. *Frontiers in Psychology,* **5**, 1-15. (2014).
